# In Situ-Derived N-Doped ZnO from ZIF-8 for Enhanced Ethanol Sensing in ZnO/MEMS Devices

**DOI:** 10.3390/molecules29081703

**Published:** 2024-04-10

**Authors:** Meihua Liang, Yong Yan, Jiaxuan Yang, Xiaodong Liu, Rongrong Jia, Yuanyuan Ge, Zhili Li, Lei Huang

**Affiliations:** 1 School of Chemistry and Chemical Engineering, Guangxi University, Nanning 530004, China; 2114391048@st.gxu.edu.cn (M.L.); 2214302082@st.gxu.edu.cn (Y.Y.); 2114391118@st.gxu.edu.cn (J.Y.); geyy@gxu.edu.cn (Y.G.); 2 Research Center of Nano Science and Technology, College of Sciences, Shanghai University, Shanghai 200444, China; 18952692333@shu.edu.cn (X.L.); r.jia@t.shu.edu.cn (R.J.)

**Keywords:** gas sensor, MEMS, ZIF-8, ZnO, ethanol

## Abstract

Microelectromechanical systems (MEMS) gas sensors have numerous advantages such as compact size, low power consumption, ease of integration, etc., while encountering challenges in sensitivity and high resistance because of their low sintering temperature. This work utilizes the in situ growth of Zeolitic Imidazolate Framework-8 (ZIF-8) followed by its conversion to N-doped ZnO. The results obtained from scanning electron microscopy (SEM) and transmission electron microscopy (TEM) indicate that the in situ derivation of ZIF-8 facilitates the adhesion of ZnO particles, forming an island-like structure and significantly reducing the interfaces between these particles. Furthermore, powder X-ray diffraction (XRD) analysis, elemental mapping, and X-ray photoelectron spectroscopy (XPS) analysis confirm the conversion of ZIF-8 to ZnO, the successful incorporation of N atoms into the ZnO lattice, and the creation of more oxygen vacancies. The ZIF-8-derived N-doped ZnO/MEMS sensor (ZIF (3)-ZnO/MEMS) exhibits remarkable gas sensitivity for ethanol detection. At an operating temperature of 290 °C, it delivers a substantial response value of 80 towards 25 ppm ethanol, a 13-fold enhancement compared with pristine ZnO/MEMS sensors. The sensor also exhibits an ultra-low theoretical detection limit of 11.5 ppb to ethanol, showcasing its excellent selectivity. The enhanced performance is attributed to the incorporation of N-doped ZnO, which generates abundant oxygen vacancies on the sensor’s surface, leading to enhanced interaction with ethanol molecules. Additionally, a substantial two-order-of-magnitude decrease in the resistance of the gas-sensitive film is observed. Overall, this study provides valuable insights into the design and fabrication strategies applicable to high-performance MEMS gas sensors in a broader range of gas sensing.

## 1. Introduction

Ethanol, a representative volatile organic compound (VOC), plays a pivotal role in various industries including petrochemicals, medical applications, and green fuels. The rapid detection of ethanol concentration is crucial for environmental and safety concerns associated with its use [1,2]. In addition, the rapid detection of ethanol is increasingly utilized in various applications, such as identifying drunk driving behavior and monitoring health indicators in daily life [3]. Metal Oxide Semiconductor (MOS)-based gas sensors have emerged as a preferred choice for rapid ethanol detection and warning systems because of their cost-effectiveness, simplicity of fabrication, swift response and recovery times, and portability [4,5,6]. However, the operating temperature of MOS-based gas sensors typically ranges from 200 °C to 500 °C, which inevitably results in high power consumption of several hundred milliwatts [7]. Recently, gas sensors based on MEMS have been at the forefront of gas sensor development, owing to their numerous advantages such as compact size, low power consumption, ease of integration, and intelligent manufacturing capabilities [7,8].

Normally, the gas-sensitive area of MEMS-based gas sensors is typically limited to tens to hundreds of micrometers, posing significant challenges for achieving precise film formation. Traditional deposition methods for gas-sensitive materials, such as drop-coating, spin-coating, and screen-printing techniques, suffer from low resolution and are challenging to accurately process on micro-hotplates [9,10,11]. Electrohydrodynamic (EHD) printing, a type of inkjet printing technology, offers high resolution with droplet sizes reaching femtoliter levels, enabling precise deposition in confined spaces [12,13]. However, the precision of EHD printing is greatly influenced by the printing needle, which often has a small aperture ranging from 20 to 200 μm [14]. This necessitates the use of a well-dispersed nanoparticle system. Because of the small size of nanoparticles, the number of interfaces between particles increases, leading to increased interfacial resistance, which affects the transmission of charges and the stability of resistive properties. As for traditional ceramic tube-based gas sensors, high temperatures are required to reduce the interface and increase the stability. Unfortunately, in the case of MEMS sensors, the allowable heat treatment temperature is often lower than 500 °C, which makes it difficult for the thin film to sinter and reduce grain boundaries [15]. This can lead to poor gas response performance and increased electrical resistance. Therefore, there is a need to explore new methods and strategies to reduce the number of nanoparticle interfaces while maintaining a high specific surface area at lower heat treatment temperatures [16].

As a typical Metal–Organic Framework (MOF), ZIF-8 is composed of zinc ions (Zn^2+^) and imidazolate ions (Im^−^), forming a stable three-dimensional porous network. Its highly tunable pores, high specific surface area, and versatility endow it with important roles in gas adsorption [17,18]. However, the weak conductivity of ZIF-8 makes it difficult to apply directly in resistive gas sensors. By deriving ZnO from ZIF-8 as a self-sacrificing template through heat treatment, porous gas semiconductor sensors can be constructed, which has attracted increasing attention [19,20]. For instance, Zhang et al. employed ZIF-8 as a sacrificial template to fabricate hollow ZnO nanocubes at 300 °C [21]. Because of the synergistic effect of their exposed surfaces and defects, these nanocubes exhibited enhanced response performance, selectivity, and a lower detection limit towards formaldehyde. Similarly, Qi et al. prepared neck-connected NC-ZnO films with controllable neck diameters by calcining core–shell ZIF-8@ZnO films at 500 °C [22]. This approach significantly improved the sensitivity, achieving a six-fold increase in response towards ethanol compared with pristine ZnO. These derivatives can be obtained at relatively low temperatures, providing a viable pathway for the adhesion of gas-sensitive particles at reduced temperatures.

Herein, we propose to in situ modify ZnO/MEMS gas sensors with ZIF-8-derived N-doped ZnO for an enhanced ethanol response. Firstly, we use EHD printing technology to print a uniform ZnO thin film on the MEMS micro-hotplate. Then, we utilize the printed ZnO as a sacrificial template for the in situ growth of ZIF-8. Subsequently, we sinter the material at 400 °C to obtain nitrogen-doped ZnO. By introducing nitrogen-doped ZnO, the gas sensitivity performance can be enhanced while adhering different ZnO nanoparticles together. This achieves a reduction in particle interfaces while avoiding a decrease in the specific surface area of the gas-sensitive film. Studies on the gas sensitivity performance of ethanol showed that the prepared ZIF (3)-ZnO/MEMS gas sensor exhibits high responsivity, particularly in detecting low concentrations of ethanol. Additionally, the resistance of the gas-sensitive film was reduced by two orders of magnitude. These improvements provide insights into obtaining high-performance MEMS-based ethanol sensors.

## 2. Results and Discussion

### 2.1. The Microstructure and Surface Properties of Materials

Figure S1 displays optical images demonstrating the transformation of sensing materials derived from in situ-grown ZIF-8 to nitrogen-doped ZnO on a ZnO/MEMS chip during various heating stages. The EHD printing technique allows for precise and controlled deposition of ZnO within a confined heating zone of 150 × 150 μm, ensuring the formation of a ZnO thin film. Following the in situ growth of ZIF-8, the film maintains its white appearance. To facilitate the observation of colorimetric changes in the gas-sensitive film during heat treatment, in situ electrical heating to 400 °C is employed. Initially, a darkening of the film is observed, which is attributed to the decomposition and carbonization of the organic ligands within ZIF-8 [23]. However, as the heat treatment progresses, the film gradually returns to a white color, indicating the completion of organic ligand decomposition and the subsequent formation of ZnO [24]. Notably, the entire transformation process occurs within a few minutes, highlighting the remarkable heating rate achieved by the MEMS-based heating system.

**Figure 1:**
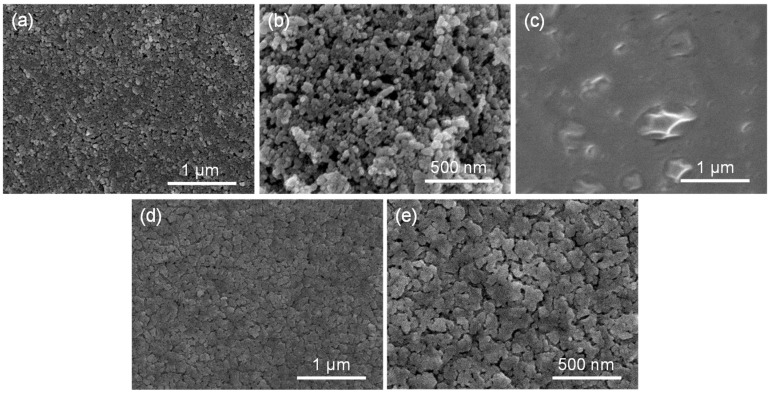
SEM images of ZnO deposited on a MEMS chip of (**a**,**b**). (**c**) SEM images of ZIF (3)-ZnO/MEMS before annealing. (**d**,**e**) SEM images of ZIF (3)-ZnO/MEMS.

The microstructure of the gas-sensitive film was investigated using SEM. Figure 1a,b indicate that the ZnO consists of irregularly shaped nanoparticles on the ZnO/MEMS chip. The surface of the ZnO film deposited via EHD printing appears relatively smooth, albeit with some inconsistencies in particle density. When the ZnO nanoparticles on the chip are used as sacrificial templates for the in situ growth of ZIF-ZnO film in a 2-methylimidazole solution, a uniform and smooth organic film is obtained (Figure 1c). Upon thermal treatment to derive ZIF (3)-ZnO/MEMS, the ZIF-8 undergoes shrinkage and collapse during pyrolysis, leading to the creation of a film with numerous gaps and a uniformly flat surface [25] (Figure 1d). Higher magnification images (Figure 1e) further confirm that individual ZnO nanoparticles are difficult to discern, as they have agglomerated into an island-like structure with tighter interparticle connections. The obtained gaps provide passageways for the diffusion of gas molecules. This agglomeration behavior is also observed in non-in situ-grown ZIF (3)-ZnO nanoparticles (Figure S2). Elemental mapping analysis further elucidates the distribution of various elements within the sample, as shown in Figure S3. The elements C, N, O, and Zn are uniformly distributed throughout the sample. Additionally, in the region of the chip where ZIF (3)-ZnO/MEMS is present, a similar elemental distribution to that of ZIF (3)-ZnO nanoparticles is observed (Figure S4), indicating the successful incorporation of N atoms into the ZnO matrix.

**Figure 2:**
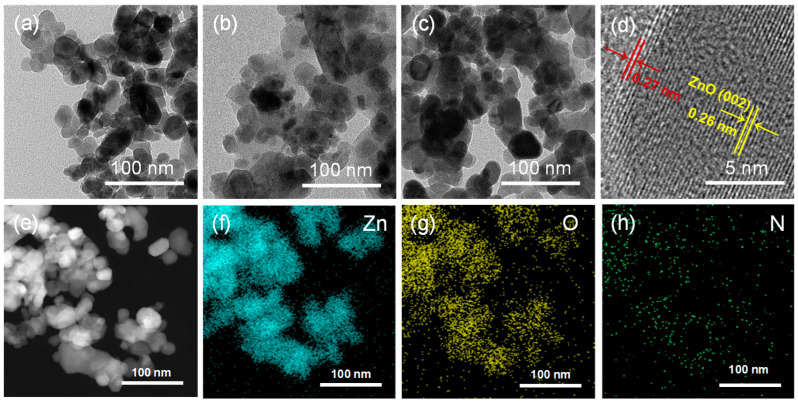
Low-magnification TEM images of (**a**) ZnO, (**b**) ZIF (3)-ZnO/MEMS before sintering, and (**c**) ZIF (3)-ZnO/MEMS; (**d**) HRTEM images of ZIF (3)-ZnO/MEMS; and (**e**) STEM and elemental mapping of ZIF (3)-ZnO/MEMS; (**f**–**h**) are the element mapping images of Zn, O, and N.

The microstructures of various materials were further studied with TEM and HRTEM. For observation, the films deposited on ZnO/MEMS, pre-sintered ZIF (3)-ZnO/MEMS, and the ZIF (3)-ZnO/MEMS sensor were detached via ultrasonic agitation and dispersed. As shown in Figure 2a, ZnO exists as irregularly shaped nanoparticles with relatively uniform dispersion and simple stacking between particles. After the in situ growth of ZIF-8 on the surface, block-like structures of ZIF-8 are formed, encapsulating the ZnO nanoparticles (Figure 2b). Following sintering at 400 °C, ZIF-8 transforms into nitrogen-doped ZnO, connecting and binding different ZnO particles together (Figure 2c). This observation aligns well with the results obtained from SEM analysis. Figure 2d presents the HRTEM image of ZIF (3)-ZnO/MEMS. The interplanar spacing of 0.26 nm corresponds to the (002) plane of ZnO [24,26]. Notably, an interplanar spacing of 0.27 nm is observed at the edges, potentially indicating the incorporation of nitrogen atoms into the ZnO lattice [27]. Figure 2f–h depict the elemental distribution maps of ZIF (3)-ZnO/MEMS. The elements C, N, O, and Zn are uniformly distributed throughout the prepared sample. This finding is consistent with the SEM elemental mapping results, further confirming the successful doping of nitrogen atoms into the ZnO derived from ZIF-8.

**Figure 3:**
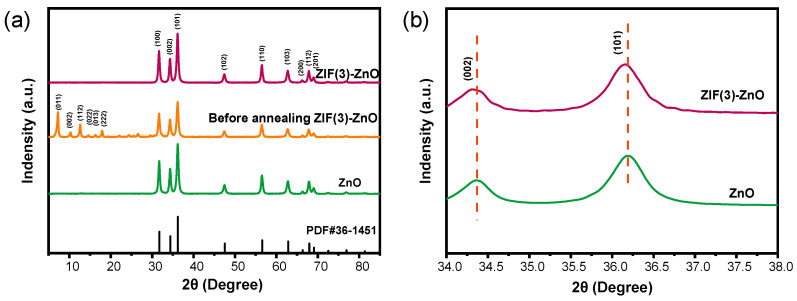
(**a**) XRD spectra of ZnO, ZIF (3)-ZnO before sintering, and ZIF (3)-ZnO; (**b**) XRD spectra of locally enlarged ZnO and ZIF (3)-ZnO.

The crystal structures of ZnO and ZIF (x)-ZnO before and after annealing were investigated by XRD. The results in Figure 3a show distinct diffraction peaks in the XRD pattern of the ZnO sample at 31.8°, 34.4°, 36.2°, 47.5°, 56.6°, 62.9°, 66.4°, 67.9°, and 69.1°, which correspond to the (100), (002), (101), (102), (110), (103), (200), (112), and (201) planes for ZnO (JCPDS No. 36-1451), respectively [28]. This indicates that ZnO possesses a typical wurtzite crystal structure. After the in situ growth of ZIF-8 on the ZnO, new diffraction peaks appeared at 7.3°, 10.3°, 12.7°, 14.6°, 16.4°, and 17.9°, which match well with the reported XRD diffraction peaks of ZIF-8 in the literature [29], confirming the formation of ZIF-8. However, following sintering treatment at 400 °C in a muffle furnace, the diffraction peaks corresponding to ZIF-8 disappeared in the XRD pattern. This suggests the complete thermal decomposition of ZIF-8 and its transformation into ZnO [27]. Similar trends were observed in XRD spectra of samples with varying ZIF-8 growth durations, as shown in Figure S5. It is worth noting that Figure 3b demonstrates a left shift in the (101) crystal plane of sintered ZIF (3)-ZnO. By employing the Debye–Scherrer formula (Equation (1)) to determine the grain size (D) and using Equation (2) to calculate the micro strain (*ε*) characterizing the degree of lattice distortion, it was found that the grain size of ZnO is 17.1 nm with a micro strain (*ε*) of 0.12%. Upon doping ZnO with nitrogen, the grain size of N-ZnO-3h decreased (*D* = 15.9 nm), while the micro strain increased (*ε* = 0.14%). According to the literature, this phenomenon could be attributed to the substitution of O atoms by N atoms or the insertion of N atoms into interstitial positions between Zn and O atoms, indicating successful doping of nitrogen into the ZnO lattice [30,31,32].

(1) $$D=K_{S}\lambda /\beta cos\theta$$

(2) $$\varepsilon =\frac{\beta }{4}tan\theta$$

**Figure 4:**
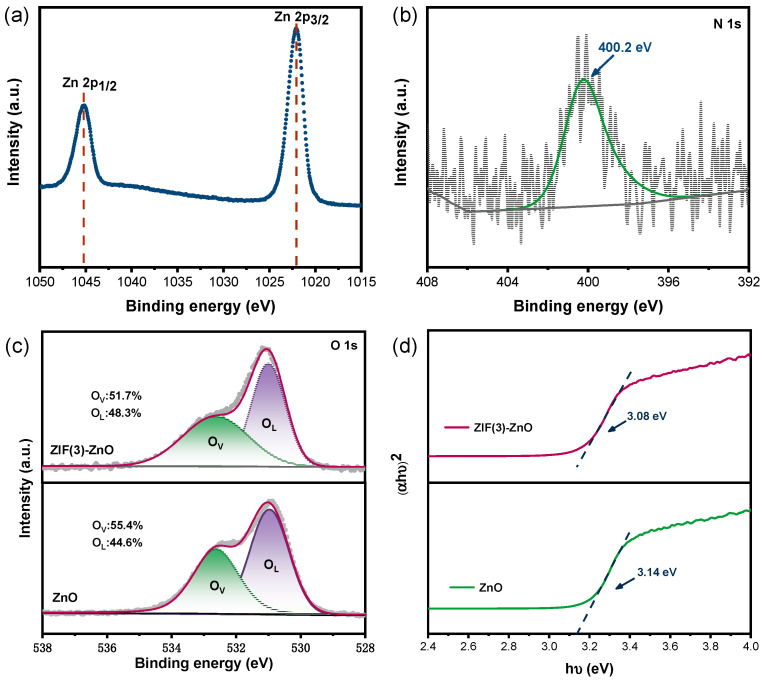
XPS spectra of ZIF (3)-ZnO samples: (**a**) Zn, (**b**) N, and (**c**) O 1s XPS spectra of ZnO and ZIF (3)-ZnO samples. (**d**) (αhν)^2^ vs. photon energy (hν) for ZnO and ZIF (3)-ZnO.

To explore the chemical valence states and compositions of Zn, O, N, and C, XPS analysis was conducted on both the ZnO and ZIF (3)-ZnO samples. Figure S6 exhibits the XPS survey spectrum of ZIF (3)-ZnO, revealing that it is composed of Zn, C, N, and O elements. The high-resolution XPS spectrum of Zn 2p (Figure 4a) shows two fitted peaks at binding energies of 1022.3 eV and 1045.2 eV, corresponding to Zn 2p3/2 and Zn 2p1/2 [33], respectively, indicating that the chemical state of Zn is primarily 2+.

Figure 4b presents the N 1s XPS spectrum of the ZIF (3)-ZnO sample. A fitted peak at 400.2 eV is attributed to the N species in the N-Zn-O bond [34], confirming the successful doping of nitrogen atoms into the ZnO lattice by substituting oxygen atoms. Figure 4c corresponds to the O 1s spectrum. According to previous reports, the binding energy of 530.9 eV can be assigned to lattice oxygen in ZnO, while 532.6 eV is associated with surface oxygen vacancies [19,27,35]. It reveals that the proportion of oxygen vacancies in ZnO is 44.6%. However, after the growth of ZIF-8 and its subsequent sintering to derive nitrogen-doped ZnO, the oxygen vacancies increase significantly, accounting for 48.3% of the total. This increase indicates that nitrogen doping favors the formation of additional oxygen vacancies on the ZnO surface.

The optical absorption properties of the ZnO and ZIF (3)-ZnO samples were investigated using UV-vis spectroscopy, and the results are shown in Figure S7 and Figure 4d. The band gap energy of the samples was calculated based on the relationship between the Kubelka–Munk function (αhν)² for direct semiconductors and the photon energy (hν). The calculated band gap for ZnO was 3.14 eV, which decreased to 3.08 eV after the growth of ZIF-8 and subsequent sintering to derive nitrogen-doped ZnO. This indicates the formation of defect energy levels (such as N-doping and oxygen vacancies) [35,36,37], consistent with the results obtained from HRTEM, XRD, and XPS.

To investigate the effect of heat treatment on the specific surface area of ZIF-8, the N_2_ adsorption–desorption properties of pristine ZnO and ZIF (3)-ZnO were studied. The isotherms are shown in Figure S8a,b. The specific surface areas calculated from these data are presented in Table S2. The specific surface area of sintered ZIF (3)-ZnO is slightly smaller at 32.7 m^2^/g compared with that of ZnO at 39.3 m^2^/g. This decrease is attributed to the collapse of ZIF-8 during the pyrolysis process, which leads to the agglomeration of ZnO particles and consequently a reduction in the specific surface area [27]. The pore size and pore volume data for ZnO and ZIF (3)-ZnO are also provided in Table S1. It is evident that the in situ growth followed by pyrolysis has minimal impact on the pore size and pore volume of ZnO.

### 2.2. The Performances of Ethanol

**Figure 5:**
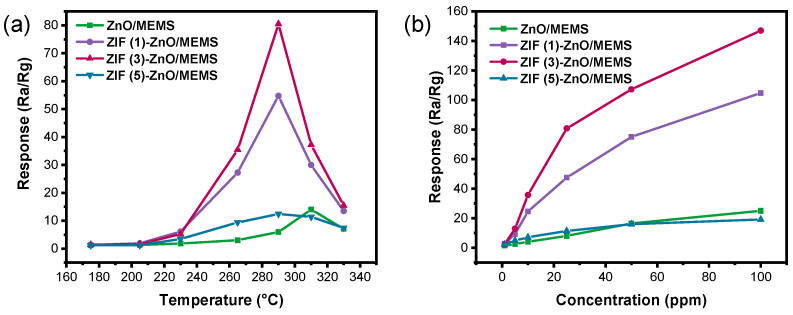
(**a**) Response to 25 ppm ethanol at different temperatures. (**b**) Response curves to 1–100 ppm ethanol at 290 °C.

Because of the significant influence of operating temperature on the gas sensing properties of MOSs, the response performance of ZnO/MEMS and ZIF (x)-ZnO/MEMS sensors towards 25 ppm ethanol was first investigated at operating temperatures ranging from 175 to 330 °C. As shown in Figure 5a, with increasing operating temperature, the response values of all four sensors exhibit a volcanic-shaped curve, initially increasing and then decreasing. Among them, the ZnO/MEMS sensor demonstrates moderate response performance, with a maximum response value of 14 at 310 °C. However, significant enhancement in gas sensing performance is observed after modification with ZIF-8. Specifically, the ZIF (1)-ZnO/MEMS sensor achieves a response value of 47.5 at an optimal temperature of 290 °C. As the growth time of ZIF-8 is extended to 3 h, the response value increases to 80.2, which is 13 times higher than that of the ZnO/MEMS sensor. Further extension of the growth time to 5 h leads to a decrease in the response value, indicating that the longer growth time of ZIF-8 is not necessarily better. Figure 5b presents the response curves of the four sensors towards ethanol concentrations ranging from 1 to 100 ppm at 290 °C. The ZIF (3)-ZnO/MEMS sensor exhibits the most superior performance among all sensors. Therefore, the ZIF (3)-ZnO/MEMS sensor is selected as the research subject in subsequent tests. The response of our sensor is compared to previously reported ethanol sensors, as shown in Table S2. Our ZIF (3)-ZnO/MEMS sensor demonstrates a higher response at lower operating temperatures than most reported materials.

**Figure 6:**
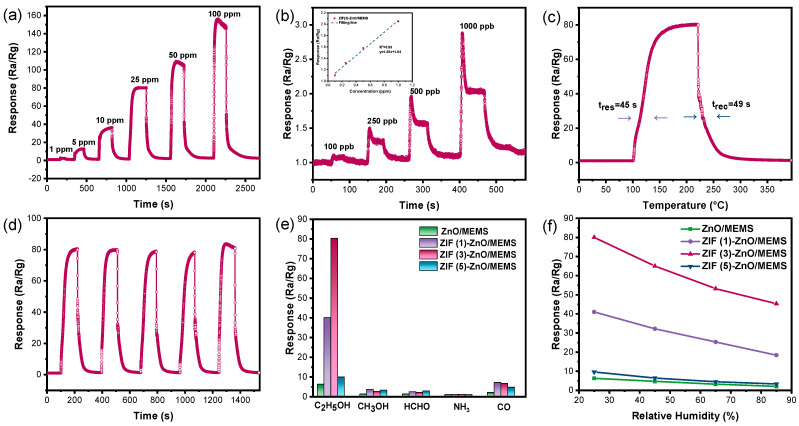
Gas sensing performance of the ZIF (3)-ZnO/MEMS sensor at a working temperature of 290 °C. (**a**) Response to 1–100 ppm of C_2_H_5_OH. (**b**) Response to 100–1000 ppb of C_2_H_5_OH along with its linearity curve. (**c**) Response and recovery time curve for 25 ppm of C_2_H_5_OH. (**d**) Repeatability test for 25 ppm of C_2_H_5_OH. (**e**) Selectivity towards different gases. (**f**) Response variation curve for 25 ppm of C_2_H_5_OH under different humidity conditions (25–85%) over different sensors.

Figure 6a presents the response curves of the ZIF (3)-ZnO/MEMS sensor to varying concentrations of ethanol. It is observed that as the ethanol concentration increases from 1 ppm to 100 ppm, the response value of the sensor also increases correspondingly. Remarkably, even at an ethanol concentration as low as 100 ppb, the ZIF (3)-ZnO/MEMS sensor maintains a notable response value of 1.13, as shown in Figure 6b. Based on the definition of the International Union of Pure and Applied Chemistry (IUPAC), the limit of detection (LOD) of the ZIF (3)-ZnO/MEMS sensor for ethanol was estimated by using the equation of LOD=3RMS_noise_/Slop. The slope of the fitting line of the ZIF (3)-ZnO/MEMS sensor is 1.03, and the root mean square (rms) noise is calculated to be 0.00396 using 30 points at the baseline without target gas. The results show that the LOD of the ZIF (3)-ZnO/MEMS sensor for ethanol is 11.5 ppb (the detailed calculation process is in the Supporting Information). These results demonstrate the exceptional sensitivity of the ZIF (3)-ZnO/MEMS sensor, even at very low concentrations of ethanol, suggesting its potential as an ultra-sensitive ethanol sensor.

Figure 6c exhibits the dynamic response curve of the ZIF (3)-ZnO/MEMS sensor at a 25 ppm ethanol concentration. With a response time of approximately 45 s and a recovery time of approximately 49 s, it demonstrates swift responsiveness. To assess the reproducibility of the ZIF (3)-ZnO/MEMS sensor, we conducted five cycles of testing, exposing the sensor to 25 ppm ethanol in each cycle (as shown in Figure 6d). The results indicate that the sensor maintains its rapid response/recovery characteristics over multiple cycles, with nearly consistent response values, thus proving its excellent reproducibility.

In practical applications, sensors are susceptible to interference from gases with similar properties, making their selectivity towards the target gas crucial in gas mixtures. To investigate this aspect, we selected several common reducing gases such as ethanol, methanol, formaldehyde, ammonia, and CO, and measured the response of the sensors to 25 ppm ethanol and the same concentration of these interfering gases at a working temperature of 290 °C. As shown in Figure 6e, the three ZIF (x)-ZnO/MEMS sensors exhibit enhanced responses compared with the ZnO/MEMS sensor across all gases tested. Notably, the response of these sensors to ethanol is significantly higher than that to other gases, indicating the excellent selectivity of the ZIF (x)-ZnO/MEMS sensors towards ethanol. Humidity is a critical factor that can affect the performance of gas-sensitive materials. herefore, we tested the response of the ZIF (x)-ZnO/MEMS and ZnO/MEMS sensors to 25 ppm ethanol under different humidity conditions (ranging from 25% to 85% relative humidity at an ambient temperature of 25 °C). As shown in Figure 6f, the response of all four sensors decreases to varying degrees with increasing humidity. However, the ZIF (x)-ZnO/MEMS sensors still demonstrate a clear advantage compared with the ZnO/MEMS sensor. These results suggest that humidity compensation may be necessary in practical applications. Overall, the findings indicate that the ZIF (3)-ZnO/MEMS sensor exhibits superior performance in terms of selectivity and humidity interference compared with the ZnO/MEMS sensor.

**Figure 7:**
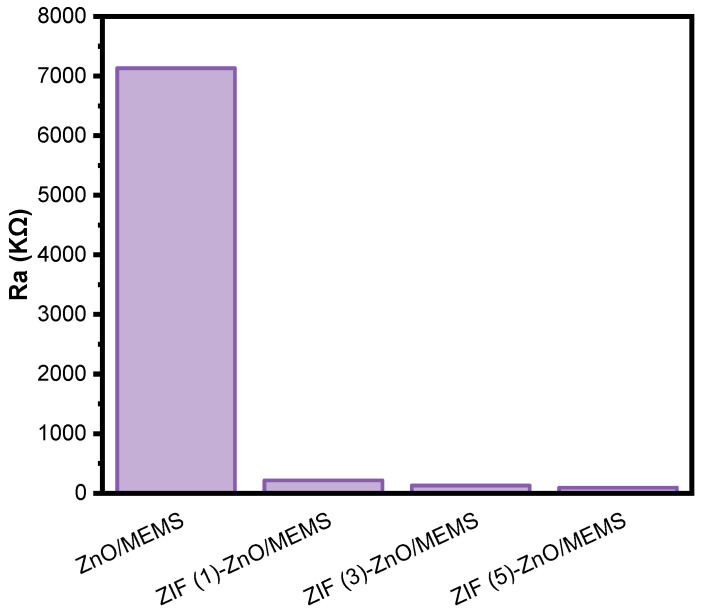
Resistance of different gas sensors at 290 °C.

Figure 7 presents the resistance (R_a_) values in the air at the optimal working temperature for the ZnO, ZIF (1)-ZnO/MEMS, ZIF (3)-ZnO/MEMS, and ZIF (5)-ZnO/MEMS sensors. The R_a_ values of these sensors follow the order ZnO/MEMS > ZIF (1)-ZnO/MEMS > ZIF (3)-ZnO/MEMS > ZIF (5)-ZnO/MEMS. Compared with the ZnO/MEMS sensor, the ZIF (1)-ZnO/MEMS, ZIF (3)-ZnO/MEMS, and ZIF (5)-ZnO/MEMS sensors exhibit a two-order-of-magnitude reduction in resistance. This reduction is attributed to the effective minimization of interface resistance between the nanomaterial particles achieved through in situ growth techniques. Interface resistance plays a crucial role in the electrical properties of gas-sensitive materials because of its close association with electron transfer at material surfaces and grain boundaries [38]. Arising from charge transfer effects at the interfaces, the reduction in interface resistance not only enhances the conductivity of the material but also reduces the barriers for electron transitions [39], thereby enhancing the gas sensing performance of the sensors to some extent.

It has been demonstrated that the sensitivity of MOS gas sensors relies on the adsorption–desorption process and the interaction of the target gas with the adsorbed oxygen anion [40,41,42]. The nature of oxyanions depends on the sensor working temperature, that is, O^2−^ (above 300 °C), O^−^ (above 200 °C and below 300 °C), and O_2_^−^ (below 200 °C) [43,44,45].

The operating temperature of this study is set at 290 °C, at which the surface oxygen species are predominantly in the form of O^−^. These anionic oxygen species accept electrons from the surface, leading to the formation of a depletion layer that subsequently reduces the conductivity of the sensor. The width of this depletion layer is directly proportional to the amount of oxygen anions absorbed on the surface. When the sensor is exposed to ethanol-containing air, ethanol molecules adsorb onto the surface and interact with the adsorbed oxygen, releasing electrons into the conduction band of the oxide sensor. This process narrows the depletion layer and enhances the surface conductivity of the sensor [44].

**Figure 8:**
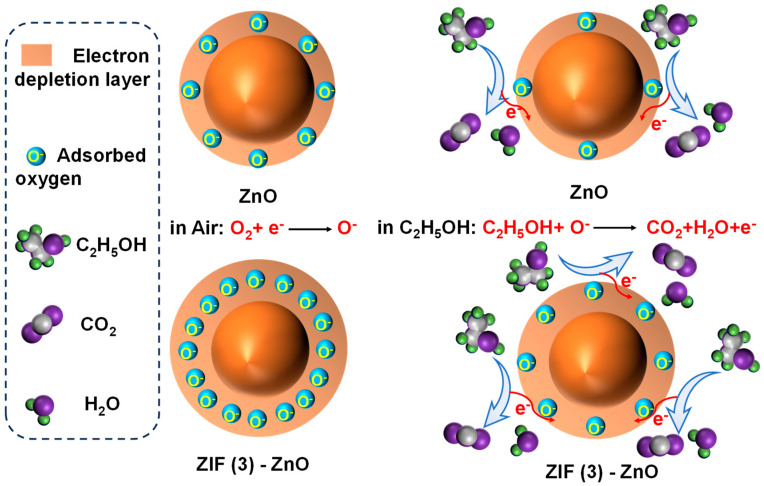
Schematic picture of the gas-sensing mechanism of ZnO and ZIF (3)-ZnO/MEMS.

In this work, the in situ growth of ZIF-8 followed by nitrogen doping introduces a significant amount of oxygen vacancies into ZnO. These oxygen vacancies facilitate the adsorption, dissociation, and ionization of oxygen molecules, resulting in the generation of more reactive oxygen species. This, in turn, enhances the interaction with adsorbed ethanol molecules, leading to a greater change in resistance before and after exposure. Consequently, the gas sensing performance is improved (Figure 8).

## 3. Experimental Methods

ZnO (50 ± 10 nm, 99.9%) was purchased from Aladdin Biochemical Technology Co., Ltd. (Shanghai, China), while 2-methylimidazole was obtained from Shanghai Macklin Biochemical Co., Ltd. (Shanghai, China) (98%). Additionally, N, N-dimethylformamide, absolute ethanol (AR, 99.7%), anhydrous methanol (AR, 99.5%), and formaldehyde solution (AR, 37.0%) were all acquired from Sinopharm Chemical Reagent Co., Ltd. (Beijing, China). All chemical substances employed in the experiments were of analytical grade and used as received without further purification.

### 3.1. Preparation of ZnO Slurry and EHD Printing

Firstly, 1 g of ZnO and 0.08 g of dispersant were added into 1.3 mL of ethylene glycol and stirred vigorously for 20 min. Subsequently, ultrasonic dispersion was performed for 2 h to obtain a ZnO slurry. MEMS devices employed were commercial ones (Figure S9), primarily consisting of interdigitated electrodes, an isolation film, a heating electrode, and a supporting film, with a gas-sensitive area measuring 150 μm × 150 μm. EHD printing was then employed on Guangdong Sygole Intelligent Technology Co., Ltd. (Dongguan, China), wherein an appropriate volume of the solution was drawn into a 1 mL syringe equipped with a needle of suitable size. It was important to ensure that no air bubbles remained within the solution as they could affect the stability of the printing process. The syringe containing the solution was mounted onto the printer’s fixture, and the needle was connected to a high-voltage power supply using a piezoelectric head. The distance between the needle and the chip was adjusted to 20 μm, and various parameters were optimized before commencing the printing process. This allowed for the uniform deposition of ZnO nanoparticles onto the MEMS chip. The printed ZnO/MEMS chip was then heated in a muffle furnace at a rate of 2 °C/min, reaching a temperature of 350 °C, where it was thermally treated for 2 h.

### 3.2. In Situ Growth of Nitrogen-Doped ZnO Derived from ZIF-8 on ZnO/MEMS Chips

**Figure 9:**
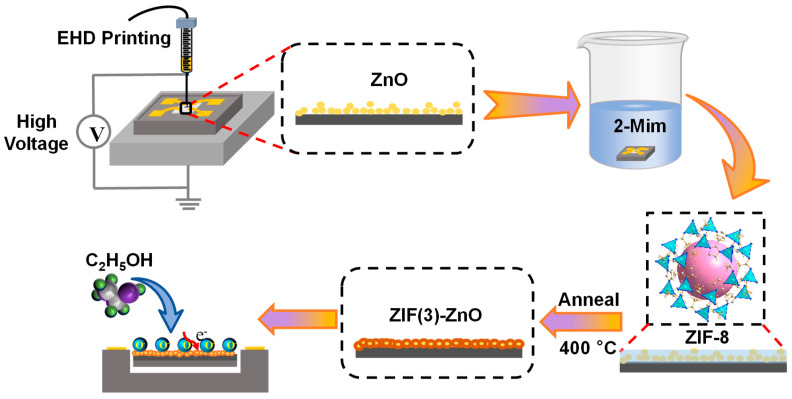
Schematic diagram of the in situ modification of ZnO/MEMS gas sensors with ZIF-8 derived N-doped ZnO.

The preparation process for the in situ growth of nitrogen-doped ZnO derived from ZIF-8 on ZnO/MEMS chips is schematically depicted in Figure 9. Initially, 0.298 g of dimethylimidazole was dissolved in a mixture of DMF/H_2_O (16 mL, 3:1 *v*/*v*) and stirred for 20 min. Subsequently, the heat-treated ZnO/MEMS chips were immersed in the dimethylimidazole solution at room temperature for a specified duration to allow for in situ growth. The chips with in situ grown ZIF-8 were then rinsed several times with ethanol and placed in an oven at 60 °C for 24 h of drying. Following this, the chips were sintered in a muffle furnace at 400 °C for 2 h, with a heating rate of 2 °C/min. The resulting materials were designated as ZIF (x)-ZnO/MEMS, where “x” represents the duration of ZIF-8 growth.

To investigate the microstructure and surface properties of ZIF (3)-ZnO/MEMS gas-sensitive materials, zinc oxide derivatives were prepared directly on ZnO nanoparticles. Under the same conditions, using DMF and H_2_O as mixed solvents, and in the presence of 2-methylimidazole, ZnO nanoparticles instead of ZnO/MEMS were employed as sacrificial templates for the growth of ZIF-8 on their surface for varying durations. Subsequently, the synthesized materials were sintered at 400 °C for 2 h to obtain ZIF (x)-ZnO nanoparticles.

The naming and definition of the samples are provided in Table S3.

### 3.3. Characterization

The crystal phase of the sample was analyzed using a X-ray diffractometer (XRD) (Rigaku, DMAX 2200, Tokyo, Japan) equipped with a Cu target (U = 40 kV, I = 40 mA). The scanning range was set from 5° to 80° with a scanning speed of 5° per minute. The morphologies and microstructure of the material were studied through scanning electron microscopy (SEM) (FEI, NOVA NANOSEM 450, Pittsburgh, PA, USA), transmission electron microscopy (TEM) (Thermo Fisher Scientific, TALOS L120C, Altrincham, UK), and high-resolution transmission electron microscopy (HRTEM) (Rigaku, JEM-2100F, Tokyo, Japan). The Brunauer–Emmett–Teller (BET) results were obtained using a fully automatic specific surface area and porosity analyzer at 77.350 K. The chemical states of surface elements were analyzed via X-ray photoelectron spectroscopy (XPS) (Thermo Fisher Scientific, NEXSA, Altrincham, UK), with the C 1s signal at 284.8 eV as the calibration standard for binding energies. The optical absorption properties of the material were investigated through ultraviolet–visible diffuse reflectance (UV-vis) spectroscopy (Agilent, Cary 5000, Hong Kong, China).

### 3.4. Gas Sensitivity Performance Test

All gas sensitivity tests were conducted on an electronic gas sensitivity tester with an intelligent analysis system (Shanghai Lingpan Electronic Technology Co., Ltd. (Shanghai, China)). Before gas sensor testing, the sensors underwent an aging process for 12 h to ensure the stability of their internal temperature and the equilibrium of surface oxygen adsorption. Apart from humidity tests, all other tests were performed under strictly controlled environmental conditions, with an ambient temperature maintained at 25 ± 5 °C and humidity kept at 25 ± 5% RH. The testing temperature range was set between 170 °C and 330 °C to comprehensively evaluate the performances at different temperatures. During the testing procedure, a micro-dosimeter or syringe was used to extract a specific amount of gas from the gas generation device and inject it into the testing chamber. By adjusting the heating voltage, we could precisely control the temperature of the chip’s central heater. The relationships between heating voltage and temperature, as well as heating voltage and power consumption, are illustrated in Figures S10 and S11, respectively.

Standard gases of CO and NH_3_ were purchased from Shanghai Weichuang Standard Gas Analysis Technology Co., Ltd. (Shanghai, China). Ethanol, methanol, and formaldehyde gases were generated using a self-made gas generation device [46] (Figure S12). The device had a heating element at its edge, which allowed for the slow addition of the liquid to be vaporized using a micropipette. This process resulted in the desired gas concentration.

To achieve varying levels of ambient humidity, a gas-sensing test chamber equipped with a heating device was employed. The response of the gas sensor to reducing gases is defined as R = R_a_/R_g_, where R_a_ and R_g_ represent the resistance of the sensor in air and the target gas, respectively. The response time and recovery time are defined as the time of 90% resistance change during the response process and the recovery process, respectively. Cross-sensitivity tests were performed using methanol, ammonia, formaldehyde, and carbon monoxide. 

## 4. Conclusions

In summary, this work demonstrates a novel approach for fabricating high-performance MEMS-based gas sensors for ethanol detection. The combination of EHD printing for controlled ZnO deposition, followed by in situ ZIF-8 growth and conversion to N-doped ZnO, offers a scalable and effective strategy. Microstructural analysis revealed that the derived N-ZnO effectively binds ZnO nanoparticles, reducing interfacial boundaries between particles and resulting in a two-order-of-magnitude decrease in sensor resistance. The ZIF (3)-ZnO/MEMS sensor exhibited a high response value of 80 towards ethanol (25 ppm) at a working temperature of 290 °C, which is 13 times higher than that of the ZnO/MEMS sensor. Additionally, it demonstrated an ultra-low theoretical detection limit of 11.5 ppb and excellent selectivity. The improved gas sensing performance is attributed to the increased concentration of oxygen vacancies induced by nitrogen doping in the ZnO lattice, which facilitates the adsorption of oxygen anions on the sensor surface, thereby enhancing the response. This work provides a strategy for the development of stable, high-performance MEMS-based gas sensors.

## Data Availability

Data are contained within the article and Supplementary Materials.

## Supplementary Materials

The following supporting information can be downloaded at: https://www.mdpi.com/article/10.3390/molecules29081703/s1. Refs. [47,48,49,50,51,52,53] are cited in the Supplementary Materials.
