# Supplementary material for: In Situ-Derived N-Doped ZnO from ZIF-8 for Enhanced Ethanol Sensing in ZnO/MEMS Devices
Source: Molecules. 2024 Apr 10;29(8):1703. doi: 10.3390/molecules29081703 (PMC11052051; doi:10.3390/molecules29081703)
Supplement: Supplementary file 1 [file molecules-29-01703-s001.zip › molecules-2953569-supplementary.pdf]

Supporting Information

In Situ-Derived N-Doped ZnO from ZIF-8 for Enhanced Ethanol Sensing in ZnO/MEMS Devices

Meihua Liang [1], Yong Yan [1], Jiaxuan Yang [1], Xiaodong Liu [2], Rongrong Jia [2], Yuanyuan Ge [1], Zhili Li [1,*] and Lei Huang [2,*]

[1] School of Chemistry and Chemical Engineering, Guangxi University, Nanning 530004, China; 2114391048@st.gxu.edu.cn (M.L.); 2214302082@st.gxu.edu.cn (Y.Y.); 2114391118@st.gxu.edu.cn (J.Y.); geyy@gxu.edu.cn (Y.G.)

[2] Research Center of Nano Science and Technology, College of Sciences, Shanghai University, Shanghai 200444, China; 18952692333@shu.edu.cn (X.L.); r.jia@t.shu.edu.cn (R.J.)

* Correspondence: lizhili@gxu.edu.cn (Z.L.); leihuang@shu.edu.cn (L.H.)

Supporting Figure

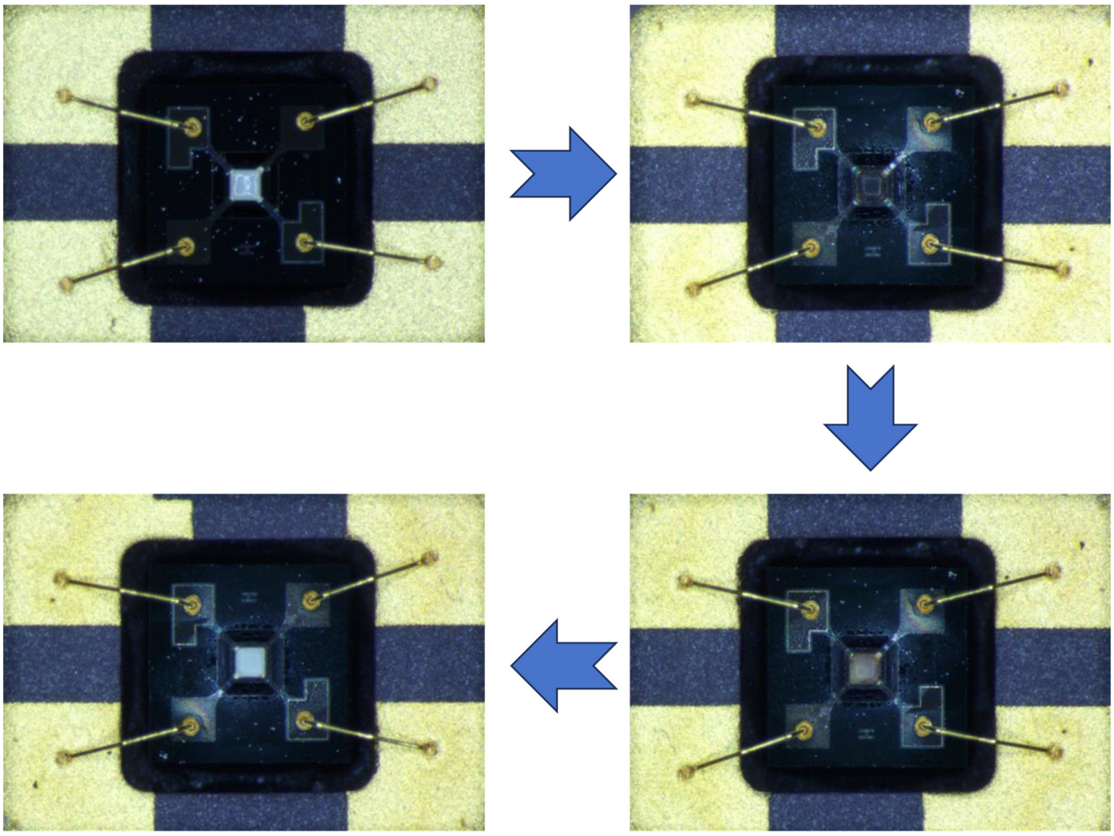

Figure S1. Photos showing the color change of the chip due to the heating of the gas-sensitive film

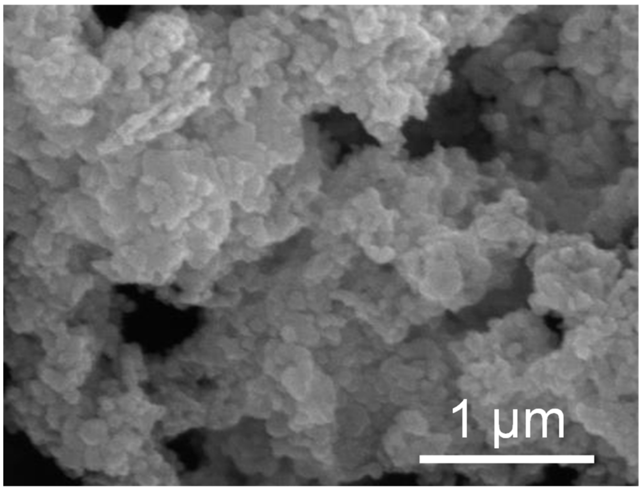

Figure S2. SEM images of ZIF (3) - ZnO nanoparticles

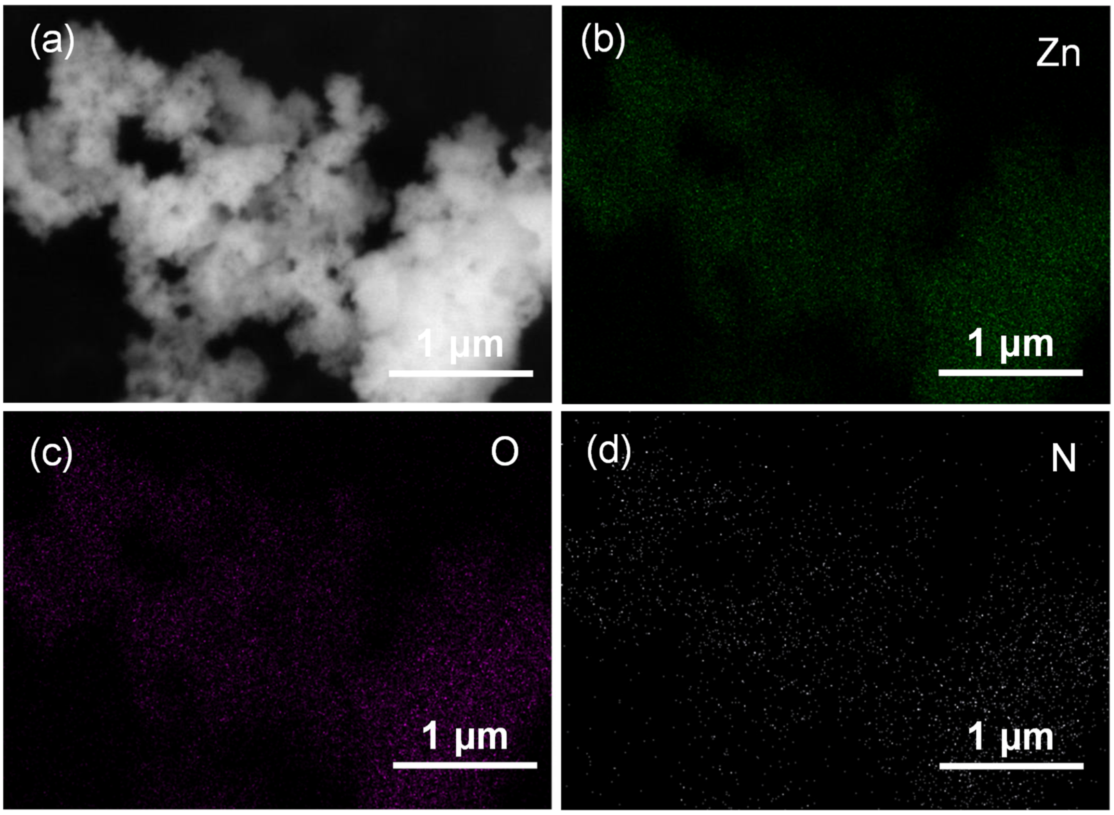

Figure S3. SEM images of ZIF (3) - ZnO nanoparticles (a), element mapping of ZIF (3) - ZnO nanoparticles with uniform distribution of C, N, O, and Zn (b-d)

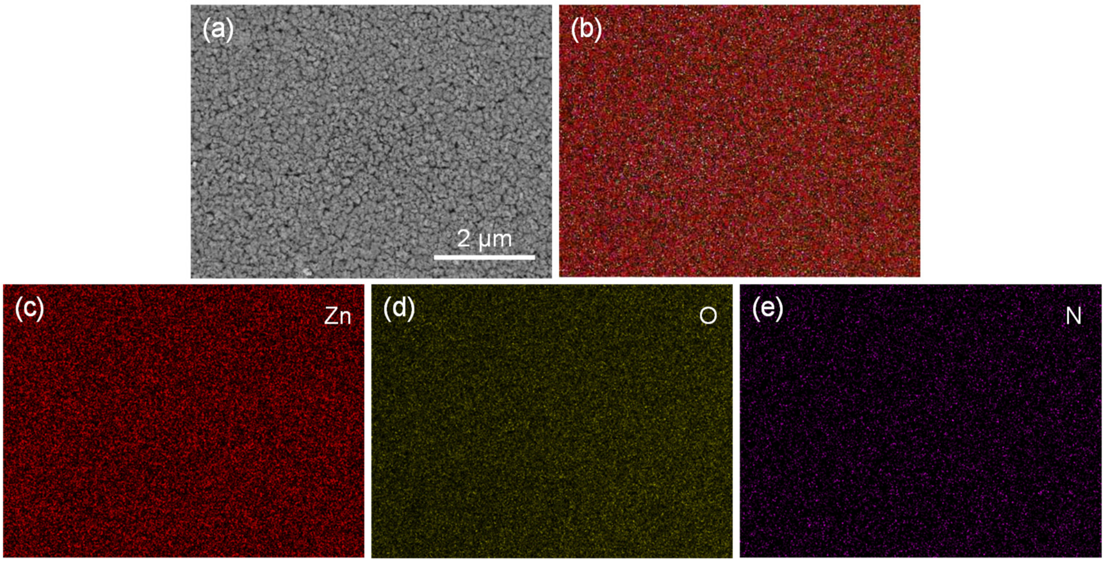

Figure S4. SEM images of ZIF (3) -ZnO/MEMS (a); C, N, O, and element mapping of uniformly distributed ZIF (3)-ZnO/MEMS (b-e)

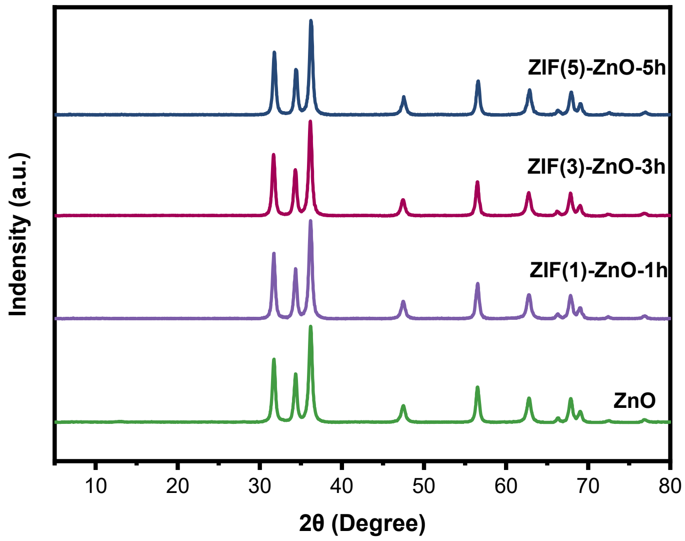

Figure S5. XRD spectra of ZnO, ZIF (1)-ZnO, ZIF (3)-ZnO, and ZIF (5)-ZnO

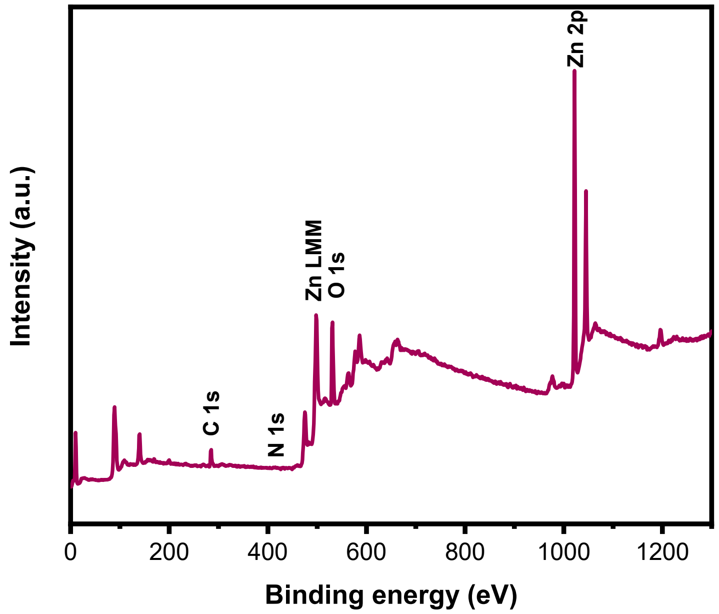

Figure S6.XPS full spectrum of ZIF (3)-ZnO nanoparticles

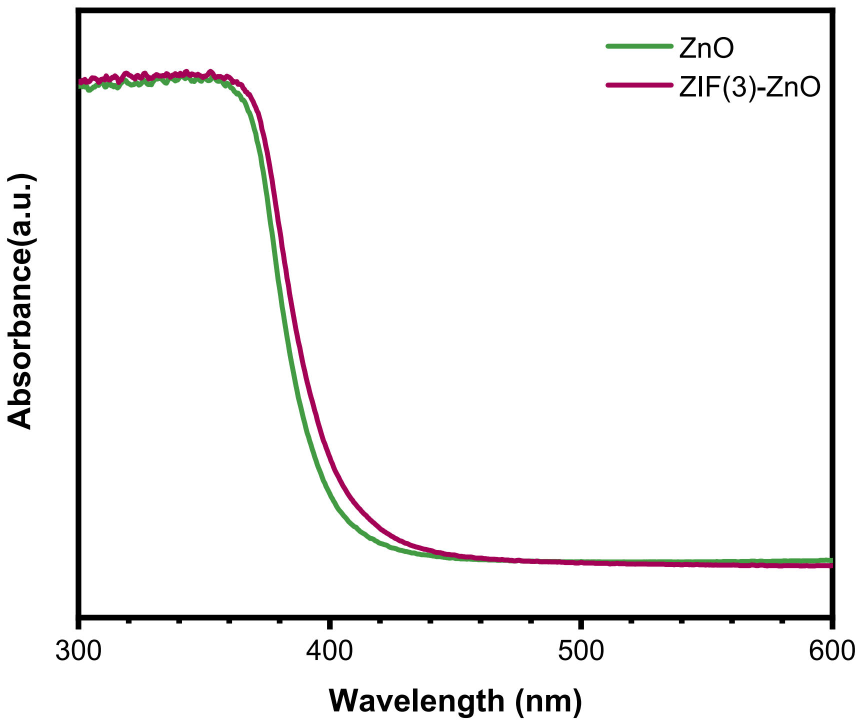

Figure S7. UV-Vis absorption spectra of ZnO and ZIF (3)-ZnO nanoparticles

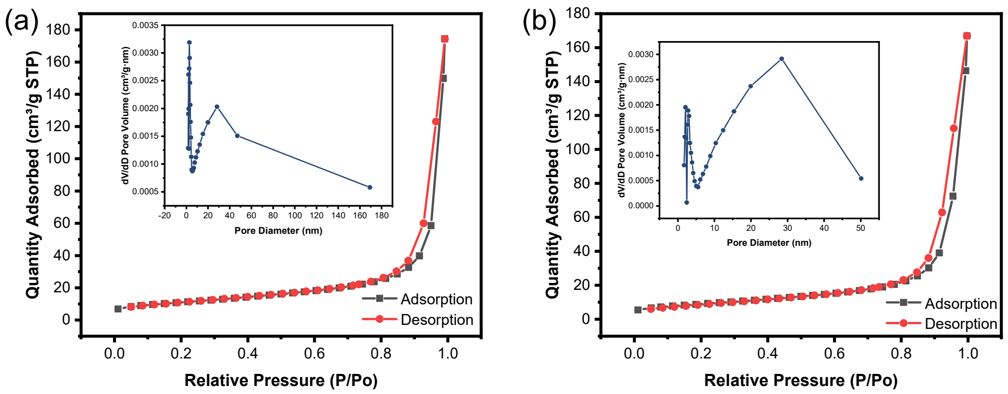

Figure S8. N_2 adsorption isotherms and pore size distributions of (a) ZnO; (b) ZIF (3)-ZnO

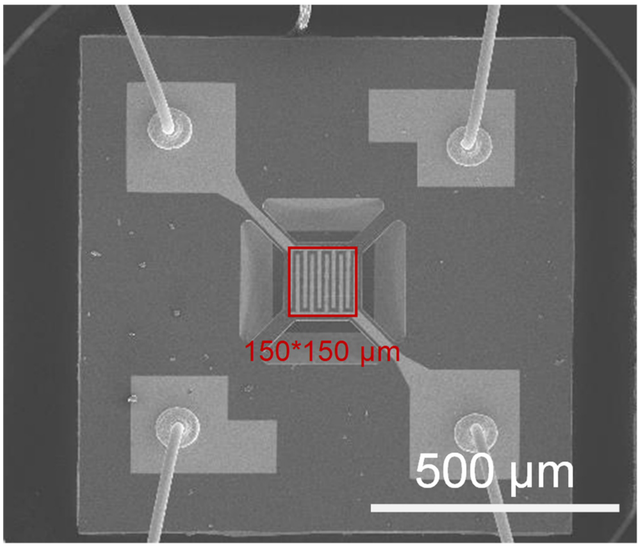

Figure S9. SEM images of blank MEMS chips

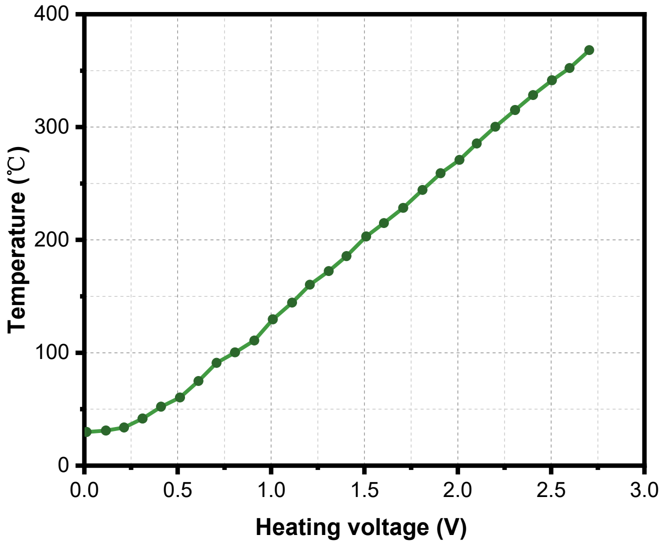

Figure S10. The relations between temperature and heating voltage

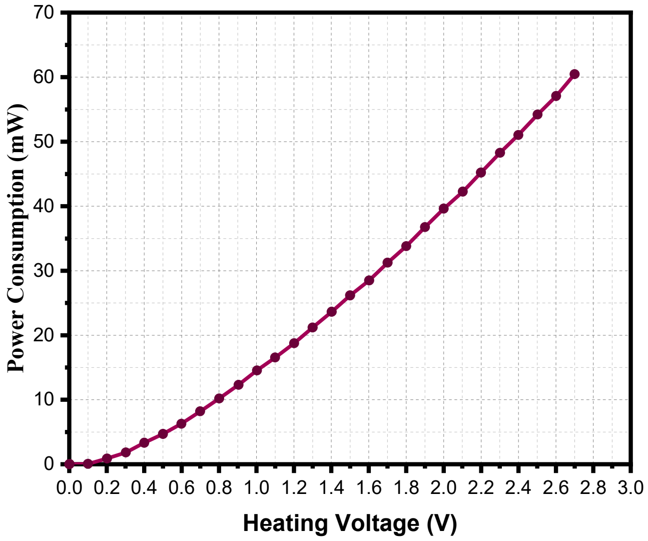

Figure S11. The relations between the power consumption and heating voltage

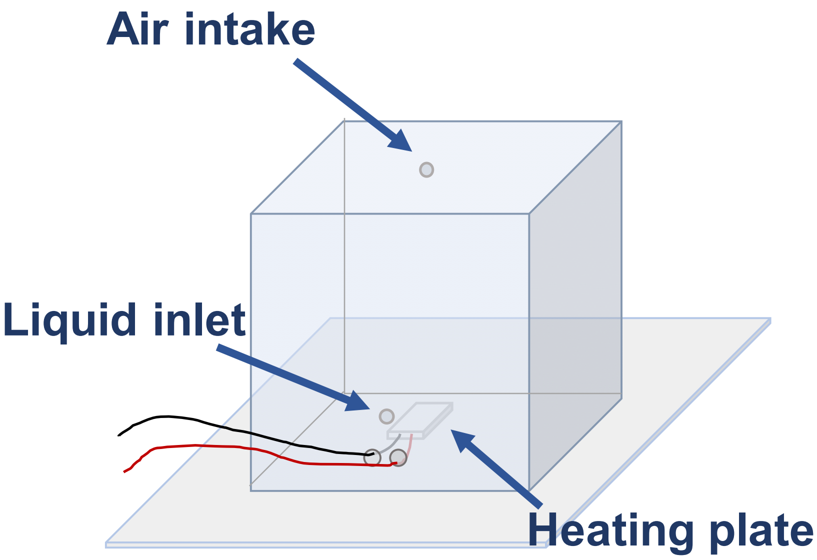

Figure S12. Gas generation device

Supporting Table

Table S1. Specific surface area, pore size, and pore volume of ZnO and ZIF (3)-ZnO

Samples	$S_{BET}(m^2/g)$	$D_{pore}(nm)$	$V_{pore}(cm^3/g)$
ZnO	39.29	27.48	0.27
N-ZnO-3h	32.74	27.69	0.26

Table S2. A comparison of the ethanol sensing performance of the sensors.

Sensing materials	$[C_2H_5OH]$ (ppm)	R_a/R_g	T_{sens} (°C)	Refs.
ZnO nanosheet	25	15	400	[1]
Double-shell ZnO hollow microspheres	100	47.4	275	[2]
HHQD-ZnO nanocages	100	139.4	325	[3]
ZnO cubes	5	1.9	400	[4]
ZnO@ZIF-8 porous nanosheets	50	58	240	[5]
ZnO@ZIF-8 core-shell material	100	35.9	160	[6]
NC-ZnO	50	124	375	[7]
N-ZnO	100	115	190	[8]
ZIF (3)-ZnO/MEMS	25	80.2	290	This work

Table S3. Definition of samples

Sample	Definition
ZnO	Untreated ZnO
ZnO/MEMS	ZnO deposited on MEMS chip
ZIF (1)-ZnO	Nanoparticles derived from the growth of ZIF-8 on ZnO nanoparticles for 1 h followed by heat treatment.
ZIF (3)-ZnO	Nanoparticles derived from the growth of ZIF-8 on ZnO nanoparticles for 3 h followed by heat treatment.
ZIF (5)-ZnO	Nanoparticles derived from the growth of ZIF-8 on ZnO nanoparticles for 5 h followed by heat treatment.
ZIF (1)-ZnO/MEMS	The ZnO/MEMS chip that underwent heat treatment after 1 h of in-situ growth of ZIF-8.
ZIF (3)-ZnO/MEMS	The ZnO/MEMS chip that underwent heat treatment after 3 h of in-situ growth of ZIF-8.
ZIF (5)-ZnO/MEMS	The ZnO/MEMS chip that underwent heat treatment after 5 h of in-situ growth of ZIF-8.

Table S4. points at the baseline without target gas

y_i	0.99259	1	0.99778	0.99111	0.99432	0.99901	1	0.99876
	0.99827	0.99827	0.99975	1.00049	1.00346	1.00569	0.99555	1.00569
	0.99852	1.00198	0.99975	1.00148	0.99506	1.00223	1.00124	1.0047
	0.99506	0.99679	1.00668	1.00396	0.99407	1.00148		

The exact procedures used for calculation of LOD.

$$\text{LOD (ppm)} = 3\frac{\text{rms}}{\text{slope}}$$

$$\text{rms}_{\text{noise}}=\sqrt{\frac{V_{x^2}}{N}}$$

$$V_{x^2}=\sum(y_i - y)^2$$

Where y_i is the measured response data at the baseline without target gas, y is the optimal response value (y=1) without target gas and the N is the number of data points. The sensor noise is y_i-y and the corresponding y_i have been shown in Table S4.

The slope of the fitting line for the ZIF (3)-ZnO/MEMS sensor was 1.03 (Figure S14)

The rms noise is calculated as $\text{rms}_{\text{noise}}$=0.00396

LOD (ppm)=3×rms÷slope=3×0.00396÷1.03=0.0115 ppm=11.5 ppb

References

1. Cao, F.F.; Li, C.P.; Li, M.J.; Li, H.J.; Huang, X.; Yang, B.H. Direct growth of Al-doped ZnO ultrathin nanosheets on electrode for ethanol gas sensor application. *Appl. Surf. Sci.* **2018**, 447, 173-181.https://doi.org/10.1016/j.apsusc.2018.03.217
2. Jiang, B.; Tao, W.; Zhao, L.P.; Wang, T.S.; Liu, X.M.; Liu, F.M.; Yan, X.; Sun, Y.F.; Lu, G.Y.; Sun, P. Double-shell ZnO hollow microspheres prepared by template-free method for ethanol detection. *Sensors and Actuators B-Chemical*. **2023**, 385, 133626.https://doi.org/10.1016/j.snb.2023.133626
3. Zhang, X.; Lan, W.Y.; Xu, J.L.; Luo, Y.T.; Pan, J.; Liao, C.Y.; Yang, L.Y.; Tan, W.H.; Huang, X.T. ZIF-8 derived hierarchical hollow ZnO nanocages with quantum dots for sensitive ethanol gas detection. *Sensors and Actuators B-Chemical*. **2019**, 289, 144-152.https://doi.org/10.1016/j.snb.2019.03.090
4. Li, W.H.; Wu, X.F.; Liu, H.D.; Chen, J.Y.; Tang, W.X.; Chen, Y.F. Hierarchical hollow ZnO cubes constructed using self-sacrificial ZIF-8 frameworks and their enhanced benzene gas-sensing properties. *New J. Chem.* **2015**, 39 (9), 7060-7065.https://doi.org/10.1039/c5nj00549c
5. Liu, T.T.; Jia, X.H.; Zhang, J.T.; Yang, J.; Wang, S.Z.; Li, Y.; Shao, D.; Feng, L.; Song, H.J. Selective detection of ethanol at low concentration by ZnO@ZIF-8 porous nanosheets. *Sensors and Actuators B-Chemical*. **2022**, 372, 132661.https://doi.org/10.1016/j.snb.2022.132661
6. Ren, G.J.; Li, Z.M.; Yang, W.T.; Faheem, M.; Xing, J.B.; Zou, X.Q.; Pan, Q.H.; Zhu, G.S.; Du, Y. ZnO@ZIF-8 core-shell microspheres for improved ethanol gas sensing. *Sensors and Actuators B-Chemical*. **2019**, 284, 421-427.https://doi.org/10.1016/j.snb.2018.12.145
7. Qi, T.J.; Yang, X.; Sun, J. Neck-connected ZnO films derived from core-shell zeolitic imidazolate framework-8 (ZIF-8)@ZnO for highly sensitive ethanol gas sensors. *Sensors and Actuators B-Chemical*. **2019**, 283, 93-98.https://doi.org/10.1016/j.snb.2018.12.010
8. Fu, H.; Feng, Z.; Liu, S.; Wang, P.; Zhao, C.; Wang, C. Enhanced ethanol sensing performance of N-doped ZnO derived from ZIF-8. *Chin. Chem. Lett.* **2023**, 34 (2), 1001-8417(2023)34:2<107425:Eespon>2.0.Tx;2-1
